# Correction: Epithelial restitution defect in neonatal jejunum is rescued by juvenile mucosal homogenate in a pig model of intestinal ischemic injury and repair

**DOI:** 10.1371/journal.pone.0212962

**Published:** 2019-02-22

**Authors:** Amanda L. Ziegler, Tiffany A. Pridgen, Juliana K. Mills, Liara M. Gonzalez, Laurianne Van Landeghem, Jack Odle, Anthony T. Blikslager

The twelfth sentence beneath the “Ussing chamber studies” sub-heading in the Methods section is incorrect. The correct sentence is: For exogenous prostaglandin experiments, 10μM 16,16-dimethylprostaglandin E_2_ was added to the basolateral chamber after the 15-minute reading for the remainder of recovery.

There is an error in the Fig 5 caption. Please see the figure and corrected caption here.

**Fig 5:**
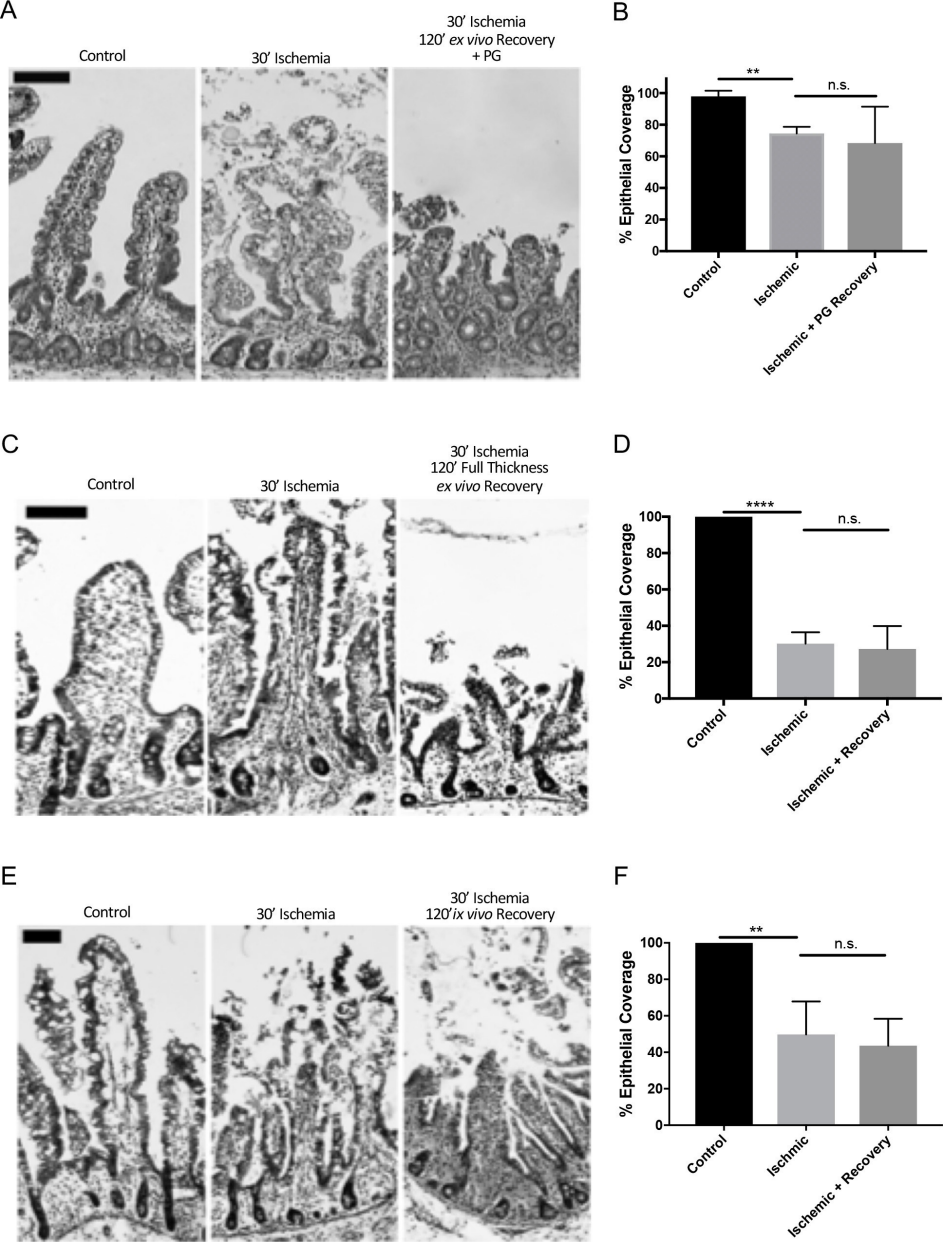
Effect of exogenous prostaglandins, full thickness *ex vivo* and *in vivo* recovery on neonatal restitution following 30-minutes of ischemia. (A) Representative histology of control, 30-minutes ischemic and 120-minutes *ex vivo* recovery neonatal jejunum with the addition of 10uM 16,16-dimethylprostaglandin E2 to the basolateral chamber. Note the persistent epithelial defect in the recovered tissue (scale bars 100 μm). (B) Histomorphometry quantified 74±2.5% and 68±13.3% epithelialization in injured and prostaglandin recovered tissues, respectively, as compared to 98±2.0% epithelialization of controls (n = 3, n.s. = not significant, **P<0.01, unpaired t-test). (C) Representative histology of control, 30-minutes ischemic, and 30-minutes ischemic and 120-minutes full-thickness *ex vivo* recovery neonatal jejunum (scale bars 100 μm). (D) Histomorphometry quantified 30±6.3% and 27±12.6% epithelialization in injured and full thickness *ex vivo* recovered tissues, respectively, as compared to 100±0.0% epithelialization of controls (n = 4, n.s. = not significant, ****P<0.0001, unpaired t-test). (E) Representative histology of control, 30-minutes ischemic, and 30-minutes ischemic and 120-*minutes in vivo* recovery neonatal jejunum (scale bars 100μm). (F) Histomorphometry quantified 50±7.4% and 44±6.6% epithelialization in injured and *in vivo* recovered tissues, respectively, versus 100% epithelialization of controls (n = 5–7, n.s. = not significant, **P<0.01, unpaired t-test).
