# Supplementary figures and images for: Epithelial restitution defect in neonatal jejunum is rescued by juvenile mucosal homogenate in a pig model of intestinal ischemic injury and repair
Source: PLoS One. 2018 Aug 23;13(8):e0200674. doi: 10.1371/journal.pone.0200674 (PMC6107120; doi:10.1371/journal.pone.0200674)

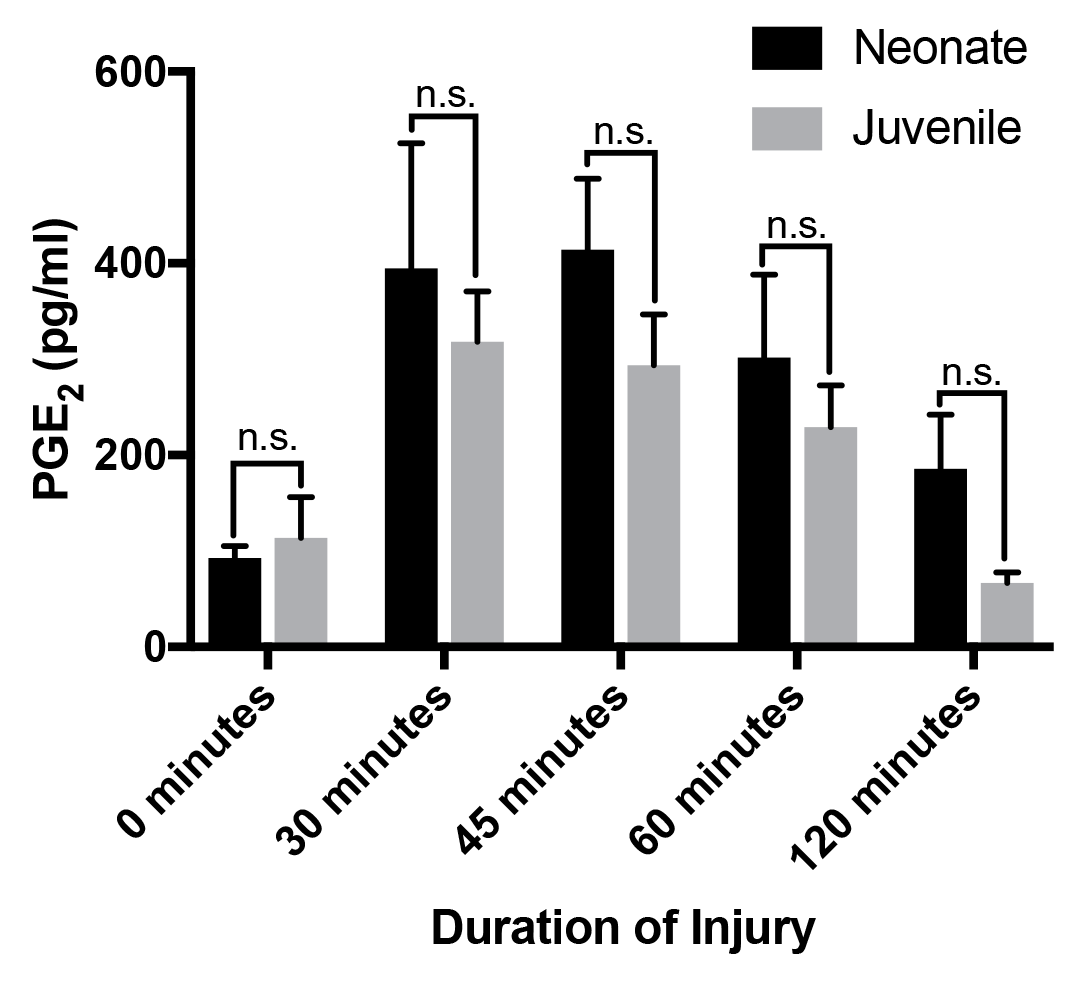

Supplement: S1 Fig — PGE2 production in the basolateral Ringer’s solution at 60-minutes of ex vivo recovery was induced by ischemic injury similarly across both age groups. (n = 5; P<0.001 for effect of injury on PGE2 by two-way ANOVA; n.s. = no significant difference on Sidak’s multiple comparisons test). (TIF) [file pone.0200674.s001.tif]
